# Perspectives and support needs for a spouse-inclusive digital intervention for perinatal depression and anxiety: a qualitative study

**DOI:** 10.4069/whn.2026.06.17

**Published:** 2026-06-30

**Authors:** Amalia Kamaruddin, Siti Roshaidai Mohd Arifin, Noor Azimah Muhammad, Mohd Said Nurumal, Hazwani Mohd Mohadis, Nik Hazlina Nik Hussain, Shanti Wardaningsih, Nur Aliya Azahari

**Affiliations:** 1 Columbia Asia Hospital Tebrau, Johor Bahru, Malaysia; 2 Kulliyyah of Nursing, International Islamic University Malaysia, Kuantan, Malaysia; 3 Faculty of Medicine, Universiti Kebangsaan Malaysia, Kuala Lumpur, Malaysia; 4 Kulliyyah of Information and Communication Technology, International Islamic University Malaysia, Kuantan, Malaysia; 5 School of Medical Sciences, Universiti Sains Malaysia, Kota Bharu, Malaysia; 6 Department of Nursing, Universitas Muhammadiyah Yogyakarta, Yogyakarta, Indonesia; 7 Klinik Anda 24 Jam Kangar, Kangar, Malaysia

**Keywords:** Anxiety, Depression, Perinatal care, Spouses, Women

## Abstract

**Purpose:**

To support digital self-management of perinatal depression and anxiety, this study explored perspectives on, and support needs for, a spouse-inclusive digital intervention among perinatal women with depression and anxiety and their spouses.

**Methods:**

The data were obtained from in-depth interviews with 20 perinatal women and 15 men who were spouses of perinatal women and attended antenatal or postnatal check-ups at selected obstetrics and gynecology clinics in Pahang and Kuala Lumpur.

**Results:**

Three main themes and seven subthemes were identified: adjusting to a new period of life, dealing with perinatal distress, and mobilizing needs and support. Perinatal women emphasized the importance of spousal support in managing emotional distress, whereas spouses described both their supportive role and the challenges they faced in providing support. Both women and spouses recommended a user-friendly online intervention for learning about maternal mental health. They identified the need for education on coping strategies, self-screening, perinatal care, childcare management, communication skills, and the signs and symptoms of depression and anxiety.

**Conclusion:**

Most women and spouses agreed that a digital intervention could help address emotional distress, depression, and anxiety, particularly because conventional approaches may be limited by high attrition rates and social barriers.

## Introduction

Women undergo substantial physical and emotional changes throughout the perinatal period, which may increase their risk of mental health problems [1]. One in five women experiences a mental health problem during the perinatal period [2]. The perinatal period is defined as pregnancy through 1 year after birth [3]. Common symptoms of poor mental health include sadness, crying easily or more often than usual, excessive worrying or “thinking too much,” reduced concentration, feelings of worthlessness, guilt, and hopelessness. Depression is a mental disorder characterized by persistent despair and loss of interest or pleasure in activities for at least 2 weeks, whereas anxiety involves feelings of tension, uneasy thoughts, and physical changes such as increased blood pressure. Physical symptoms may include shaking, sweating, light-headedness, and a rapid heartbeat [4].

Perinatal depression and anxiety not only affect maternal functioning but may also influence birth outcomes, breastfeeding, mother–infant interactions, and child growth and development [5]. Several factors have been associated with perinatal depression and anxiety, including lower educational attainment, younger maternal age, smoking during pregnancy, a history of depression, low family economic status, poor marital relationship quality, adverse life events, antenatal depression and anxiety, and lack of social support [6].

Previous studies have reported that lack of social support, especially from spouses, can contribute to perinatal depression and anxiety [7,8]. Correspondingly, spousal involvement has been recognized as a potentially protective factor against mental health problems during the perinatal period. Efforts to address perinatal depression and anxiety should therefore include strategies to strengthen this support [9-11]. Alternatives to clinic-based or health professional-led interventions should also be explored. Digital interventions have been reported to improve perinatal depression and anxiety, particularly in Western countries [12-14].

Conventional health visitor-delivered screening has several limitations, including limited staff time, parental reluctance to disclose mental health difficulties to professionals, lack of spouse screening, and language barriers identified during digital screening implementation [15]. However, many existing digital interventions for perinatal mental health are designed primarily for women and do not actively involve spouses [16]. In addition, the absence of an explicit theoretical or digital health framework in some interventions may affect implementation and long-term sustainability [17].

Women may characterize perinatal mental health problems as personal issues and often try to manage them on their own through social networking sites rather than seeking professional assistance [18]. Therefore, digital self-management with spousal support may have considerable potential for addressing perinatal depression and anxiety. This study aimed to explore perspectives on, and support needs for, a spouse-inclusive digital intervention for perinatal depression and anxiety among women with depression and anxiety and their spouses. Understanding these experiences and needs may inform the development of a digital intervention that is responsive to the realities of perinatal women and their spouses.

## Methods

**Ethics statement:** This study was approved by the Kulliyyah of Nursing Post-Graduate and Research Committee (KNPGRC) (IIUM/313/14/3/1), IIUM Research Committee (IREC) (IIUM/504/14/11/2/IREC 2022-139), and Ethics Committee for SASMEC (IIUM/413/013/14/11/2/IIR22-35) and HCTM (UKM PPI/111/8/JEP-2022-707). Informed consent was obtained from the participants.

### Study design

A generic qualitative approach was used to understand the experiences and perspectives of perinatal women with depression and anxiety and their spouses across different social backgrounds in Malaysia. The main aim of a generic qualitative design is to discover and understand a phenomenon, process, or the perspectives and worldviews of the people involved [19]. Generic qualitative research is also useful because it is sufficiently adaptable to be modified as new data and insights emerge [20].

A generic qualitative approach was considered the most appropriate method for gathering information on the perspectives, preferences, and informational or educational needs of perinatal women and their spouses, rather than generating theory or capturing the essence of lived experience, as required in grounded theory or phenomenology, respectively. This approach supported the pragmatic objective of integrating the findings into an applied framework for digital self-management of perinatal depression and anxiety. It also enabled flexible and open exploration of these issues, allowing perinatal women and their spouses to describe their views in a context-sensitive and practice-oriented manner aligned with the study aim of producing actionable knowledge for intervention development. The Consolidated Criteria for Reporting Qualitative Research Studies served as a reference for the methods and findings reported in this article [21].

### Study participants

Purposive sampling was used to recruit participants based on the following criteria: (1) aged 18 to 45 years; (2) pregnant or 4 to 52 weeks postnatal at screening; (3) Malaysian nationality; (4) able to speak Malay or English; and (5) at risk of perinatal depression or anxiety, defined as an Edinburgh Postnatal Depression Scale (EPDS) score ≥12 [22] or a Depression Anxiety Stress Scale-21 (DASS-21) score ≥8 for depression or ≥7 for anxiety [23]. The required sample size for a qualitative study depends on the achievement of data saturation. Previous studies have reported that the median number of interviews needed to reach data saturation is 15 to 30 [24]. In this study, data saturation was achieved between interviews 25 and 30, when no new themes or meaningful insights emerged from subsequent interviews.

### Research instrument

During screening, perinatal women with symptoms of antenatal or postnatal anxiety and depression were assessed using the DASS-21 and EPDS. Because the questionnaires measure different components of mental health, women were screened during both the antenatal and postnatal periods for signs of anxiety and depression. The EPDS measures depressive symptoms, whereas the DASS-21 measures depression, anxiety, and stress symptoms and can be used with perinatal women [25]. The EPDS includes general items that can be used not only for postnatal women but also for antenatal women. In addition, some EPDS items are not included in the DASS-21.

In-depth interviews were conducted to explore couples’ perspectives, preferences, and informational or educational needs regarding spouse-inclusive and digital interventions for managing perinatal depression and anxiety. A topic guide, developed from a systematic review and the research team’s perspectives, was used to facilitate the interviews. In-depth interviews were chosen because they enabled participants to describe their feelings and provide detailed accounts of their experiences with depression and anxiety.

### Researchers’ preparation

A total of 35 participants, comprising 20 women and 15 spouses, were interviewed by the first author, a Master’s-level researcher in nursing science with formal training in qualitative research methods and prior experience conducting in-depth interviews in maternal mental health settings. The research team included multidisciplinary researchers in nursing, medicine, information and communication technology, psychology, and maternal health from local and international institutions. All researchers contributed throughout the research process, including conceptualization, formal analysis, manuscript preparation, and critical review. The team had academic and clinical expertise relevant to perinatal mental health, digital health interventions, qualitative inquiry, and maternal care. Before data collection, the research team discussed the study objectives, interview procedures, ethical considerations, and reflexivity. The interviewer was female, which helped build rapport and encouraged participants to share their experiences and perspectives on perinatal depression and anxiety openly.

### Procedures

A total of 35 participants, including 20 women and 15 spouses, were interviewed by the first author. The two participant groups were perinatal women with anxiety and depression and their spouses. Screening and interviews formed the initial stages of recruitment for perinatal women. Women were approached at the obstetrics and gynaecology (O&G) clinic at Sultan Ahmad Shah Medical Centre, International Islamic University Malaysia; the O&G clinic at Hospital Canselor Tuanku Muhriz, Universiti Kebangsaan Malaysia, and the family health clinic during antenatal and postnatal visits. Data were collected between September 2023 and March 2024, and each in-depth interview lasted approximately 45 minutes to 1 hour.

Perinatal women who met the eligibility criteria and showed signs of anxiety or depression were identified during antenatal or postnatal visits using the EPDS and DASS-21 questionnaires. The researcher invited eligible women to participate in the study and provided a consent form and participant information sheet. Participants were then invited to an in-depth interview, which was scheduled either for a later time convenient to them or on the day of screening if both the researcher and participant agreed.

Each perinatal woman was contacted individually and given the option to participate or decline. After agreement was obtained, interview sessions were arranged according to the women’s preferences. A total of 20 perinatal women participated in this study. Depending on participant preference, as specified in the interview guide, interviews were conducted individually either in a private room at the clinic or virtually. Some employed perinatal women preferred weekend interviews because they were busy on weekdays, and the researcher made efforts to avoid interrupting their working hours.

A digital voice recorder and smartphone voice recorder were used to record interviews with the participants’ consent. All interviews were conducted in Malay, with some English used throughout. Field notes were recorded as soon as feasible after each interview. Participants’ gestures and facial expressions during the interviews were also documented. The field notes also included additional problems or issues that arose and affected the interview.

Spouses of perinatal women were invited to participate while waiting for their wives at the clinic. The study objectives and protocol were explained thoroughly, and agreement to participate in the interview was then obtained. Spouses were given sufficient time to consider participation. Those who agreed received a consent form, research information sheet, and interview appointment according to their convenience and the researcher’s availability. Perinatal women and spouses were informed that participation was voluntary and that they could participate or withdraw from the study at any time without penalty. Finally, participants were briefed on the potential risks and benefits of taking part in the study.

To reduce bias, perinatal women and their spouses participated in separate interview sessions. This allowed the women to discuss their concerns and experiences freely without spouse influence. While waiting for the women to complete their medical check-ups, some spouses chose to be interviewed in person at the clinic, whereas others preferred online interviews through Google Meet (Google LLC, Mountain View, CA, USA). When participants brought children to clinic interviews, interruptions sometimes occurred. Probing was used throughout the interviews to clarify issues and elicit more detailed information. Interviews were conducted using open-ended questions and a topic guide.

The interview guide was developed based on a systematic review and included core questions with structured protocols for perinatal women and their spouses, along with open-ended probes for further exploration of participants’ experiences and perspectives. For example, perinatal women were asked, “Could you share with me what you understand regarding mental health during pregnancy?”, “What information could be helpful to improve your mental health during pregnancy?”, and “Are you familiar with any digital app? What is your opinion on whether there is a digital intervention related to mental health for perinatal women?” Spouses were asked, “Could you share your experience of caring for your wife throughout her current pregnancy and after childbirth? If so, how?” and “What is your opinion on a digital intervention related to mental health for perinatal women?” The guide was used flexibly during the initial interviews to support data collection, and minor refinements were made after early interviews to improve clarity and participant understanding.

During the interviews, the researcher began with simple questions before progressing to more sensitive topics and used probes when needed to encourage elaboration. The same researcher conducted all interviews with perinatal women and spouses, and field notes were taken immediately after each session to document non-verbal cues, such as facial expressions and emotional tone. Participant information, including non-identifiable codes or pseudonyms, age, and details of the date and place of data collection, was recorded in a recruitment log to ensure confidentiality and transparency. At the end of each interview, participants were thanked for their time and invited to ask questions about the study.

### Data analysis

Data analysis was conducted manually, with all transcripts managed, coded, and organized by the researcher using a systematic framework analysis approach to ensure familiarity with and close engagement with the data. Framework analysis, consisting of three stages, namely data management, descriptive accounts, and explanatory accounts, was used to analyze the interviews [26]. The data management stage included data familiarization and careful selection of the transcripts to be evaluated. Interview recordings were transcribed verbatim and repeatedly read to achieve data familiarization. During this stage, preliminary ideas and patterns were noted. Open coding was then conducted by identifying meaningful data segments and assigning initial codes that reflected participants’ responses. These codes were derived inductively from the data and informed by the study objectives. The initial coding framework was then developed and applied across transcripts, followed by constant comparison to refine, merge, or expand codes as necessary.

The thematic framework was examined across the descriptive accounts to identify connections and similarities among categories. Data coding was conducted by a single researcher and reviewed by three members of the research team. Any discrepancies were resolved through discussion to ensure consistency. Related codes were grouped into categories, which were then organized into broader themes that captured shared patterns across participants. The analysis involved continuous movement between the data, codes, and emerging themes to ensure coherence and consistency. Finally, the explanatory accounts involved examining the distribution and degree of correspondence among phenomena across the full data set. The themes were reviewed, defined, and clearly labeled to represent participants’ perspectives accurately while ensuring that the findings remained grounded in the original data and aligned with the study aims.

### Rigor

Reflexivity was incorporated to acknowledge the researcher’s position and its potential influence on the research process. Reflexivity was treated as an ongoing practice throughout data collection and analysis, whereby the researcher critically examined how her background, values, and interests might shape the study. Before the research began, the researcher reflected on her academic background and personal motivation for selecting the topic, particularly her interest in perinatal depression and anxiety, which emerged from observations of women in the Malaysian context and was supported by existing literature on its effects on maternal and infant well-being. These reflections allowed the researcher to recognize assumptions that could influence the direction of the study. During data collection, participants were encouraged to share their experiences openly. However, some expressed hesitation because of the sensitive nature of depression and anxiety. The researcher maintained a neutral and empathetic stance, drawing on her background as a healthcare student to understand participants’ situations while ensuring that responses were not shaped by her expectations.

Confidentiality was emphasized, and pseudonyms were used to encourage openness. Field notes were recorded after each interview to document non-verbal cues, contextual observations, and significant interactions. During data analysis, reflexivity was maintained through continuous reflection and documentation of ideas and emerging interpretations. Reflective notes were used to examine how themes were developed and to minimize potential bias. The analysis process was further supported by regular discussions among the co-authors. Emerging codes, categories, and themes were reviewed collaboratively by the research team to enhance rigor and credibility. These practices helped ensure that the findings remained grounded in participants’ accounts and strengthened the overall trustworthiness of the study.

## Results

Table 1 shows the demographic characteristics of perinatal women and their spouses. Participants were 25 to 40 years old. Most participants in both groups had attained tertiary education. Among the perinatal women, 13 were employed and seven were unemployed. In contrast, most spouses were employed in the government or private sector, with a smaller number being self-employed. Most women were in the antenatal phase, whereas only a few were in the postnatal period.

Table 2 presents the comparative thematic analysis of interviews with perinatal women and spouses across three themes and seven subthemes. The first theme, adjusting to a new period of life, comprised emotional distress, psychological distress, and social distress. The second theme, dealing with perinatal distress, included subthemes related to support systems and stress-relieving strategies. The third theme, mobilizing needs and support, encompassed approaches to digital applications and beneficial content required for intervention. Both groups described the transition to parenthood as a period of adjustment; however, perinatal women consistently reported more pronounced emotional, cognitive, and physical distress, including feeling that life was not normal, mental preoccupation, cognitive difficulties, and physical discomfort affecting daily functioning. In contrast, spouses tended to focus less on internal psychological experiences and more on external roles, responsibilities, and practical aspects of adaptation.

In relation to coping with perinatal distress, perinatal women emphasized the importance of social and emotional support, particularly active spousal participation and support from family and friends, along with positive coping strategies such as relaxation and self-care. Spouses acknowledged their responsibility to provide support but also described perceived limitations in fulfilling this role and provided less detail about personal coping strategies. For mobilizing needs and support, both groups expressed a preference for user-friendly digital interventions; however, perinatal women prioritized interactive features and awareness-building functions, whereas spouses highlighted the need for practical guidance to better understand and enact their supportive role. Across both groups, participants converged on the need for psychoeducation, particularly regarding perinatal mental health, symptom recognition, and effective coping and communication strategies, although the specific focus differed by role.

### Theme 1: adjusting to a new period of life

When describing changes during the perinatal period, perinatal women and their spouses reported several adjustments related to emotional distress, including increased sensitivity, frequent crying, irritability, and other emotional changes. These changes were reported by several perinatal women, as shown below:

“During my first pregnancy, I would say that, about once a month, I cried and did not know why. I am not usually the type of person who cries easily, but when I was pregnant, I suddenly cried every night before going to sleep. I was not just crying a little; I cried so badly that even I did not know what was wrong with me. I just wanted to cry during that time...” (W004).

In addition to crying without a clear reason, one antenatal mother reported that she became easily irritated and inactive, choosing to stay in her room all day during her second pregnancy.

Perinatal women described feeling different during the perinatal period because it was a period of adjustment in many aspects of life, especially emotionally. They reported that they did not feel like themselves and could not think clearly during this time. Similarly, one spouse described comparable changes in his wife during the interview, as shown below:

“Her emotions were easily affected and unstable after childbirth. In addition, her thoughts were not clear, and she was sometimes restless” (S005).

In addition to recognizing changes in personality, perinatal women and spouses also reported that women were prone to overthinking during the perinatal period. Overall, emotional distress was common among perinatal women during the antenatal and postnatal periods when emotional disturbances occurred. Some women reported that these changes made them feel useless and unlike themselves compared with their usual routines, as stated below:

“So, overthinking is another reason that can lead me to anxiety because I am always thinking about whether I can breastfeed or take care of this baby afterward. Thinking too much makes me feel anxious” (W005).

In addition, almost all perinatal women and spouses reported that psychological distress, including changes in cognitive functioning and physical discomfort, was an indicator of perinatal depression and anxiety. Changes in cognitive functioning were reflected in behaviors that differed from women’s usual routines during pregnancy and after childbirth. One spouse reported that his wife’s behavior became unusual, as shown below:

“I noticed that when my wife was pregnant with our third child, she talked to herself. I felt strange at that time seeing her talking to herself like that” (S011).

One of the antenatal women also pointed out:

“After giving birth, we should take care of our emotions and should not get angry, even when we see that the house is messy; we should just ignore it first. Those things can lead to anger and make us end up thinking too much because we keep thinking that this or that work is unfinished. Those things make us feel stressed” (W017).

In addition to changes in cognitive functioning, some participants reported physical discomfort, such as sleep difficulties, restricted movement, loss of appetite, and fatigue, resulting from bodily changes during the perinatal period. One participant described sleep-related fatigue as follows:

“When the baby stays up at night, I cannot sleep properly. Because of that, I get tired easily and start feeling very stressed. I felt stressed because of lack of sleep and because I needed to take care of my baby alone without my spouse’s participation” (W010).

In addition, one spouse reported that his wife had difficulty moving after cesarean section and that this restriction affected her emotions:

“In my situation, my wife had movement restrictions because of the cesarean section, so it was hard for her to get up, and she moved slowly. If the baby started crying, it would affect her emotions in that situation” (S007).

Some perinatal women experienced physical discomfort, such as shortness of breath, during pregnancy, as shown below:

“During pregnancy, I always experienced shortness of breath, especially when talking, and hand numbness that worsened at night. I needed to use two pillows when sleeping to feel more comfortable” (W003).

Similarly, one antenatal mother reported shortness of breath during her second pregnancy. These accounts show that physical discomfort, including breathlessness, palpitations, and difficulty sleeping, was experienced by many antenatal women during pregnancy.

Social distress included long-distance relationships (LDRs), financial problems, childcare burden, workplace stress, and lack of spousal support, all of which contributed to emotional distress among perinatal women. Spouses also reported that an LDR was one contributing factor that could lead to depression and anxiety.

“An LDR with my wife is one of the factors that can lead her to anxiety because I return home every 2 days. Being alone at home during pregnancy can increase her anxiety level” (S001).

Perinatal women reported feeling alone during pregnancy because they did not receive sufficient attention from their spouses due to LDRs. They had to go through pregnancy without their spouses being present all the time.

In addition, spouses reported that workplace stress could also contribute to depression and anxiety.

“Sometimes, workplace stress can be one of the factors that leads my wife to anxiety. For instance, a lot of work needs to be done, especially before delivery, because too much work has been delayed” (S007).

In addition to workplace stress, financial issues were reported as factors contributing to depression and anxiety. Spouses acknowledged that financial constraints could contribute to emotional disturbance.

“For me, financial problems were one of the contributing factors that caused emotional distress” (S003).

Perinatal women and spouses also struggled with adaptation to parenthood, especially if they were first-time parents. First-time parents often lacked knowledge and experience in caring for a newborn, which affected their emotions during the perinatal period, particularly after childbirth. Most women reported feeling burdened by childcare and lacking knowledge about newborn care. W008 stated that she felt anxious because she did not know how to care for a newborn, as this was her first pregnancy.

“I also feel anxiety because this is my first child, and I have no childbirth experience, so the anxiety increases, especially as the due date approaches. I fear how childbirth will go, whether I can have a normal delivery, and whether I will be able to breastfeed like others when the baby arrives” (W008).

Similarly, one spouse reported that his wife experienced anxiety because she lacked knowledge about caring for the baby.

“My wife feels anxiety because this was our first child, and she did not know how to push during delivery or breastfeed the baby correctly” (S001).

Most perinatal women and spouses indicated that they needed to adjust to a new period of life while facing three types of change: emotional, psychological, and social distress. These changes contributed to their experiences of depression and anxiety during the perinatal period.

### Theme 2: dealing with perinatal distress

In addition to adjusting to a new period of life during the perinatal period, perinatal women described how they dealt with perinatal depression and anxiety through support systems, including spouses, family, and friends, as well as various stress-relieving strategies. These strategies were perceived as helpful for managing responses to emotional distress, as expressed below:

“My spouse supports me by listening when I share my problems with him. Sometimes, he lets me rest completely and does the household chores if I feel tired. That is how he shows me that he supports me throughout my pregnancy journey” (W007).

In addition to spousal support for reducing perinatal depression and anxiety, one postnatal mother stated that her family, especially her mother and sister, always helped her manage the baby and provided considerable advice during the postnatal period. This support helped reduce her depression or anxiety levels during the postnatal period and allowed her to take short breaks to rest.

The descriptions above indicate that perinatal women preferred to share emotional distress with the people they trusted most, including family members and friends, as a way to reduce perinatal depression and anxiety. In addition to having a strong support system, one perinatal woman suggested seeking help from healthcare professionals (HCPs) when dealing with stress, as described below:

“When we experience emotional distress or inner problems, we need to seek help from healthcare providers to solve our problems and reduce depression and anxiety” (W013).

Similarly, W006 learned to manage her emotions through breathing exercises and other activities that diverted her attention and reduced depression and anxiety during pregnancy.

Many perinatal women also reported that sharing problems with their spouses could alleviate stress. They took positive action by sharing their concerns with their spouses, as stated below:

“When I have problems at my workplace, I like to share them with my spouse so that my stress can slowly decrease” (W001).

“Usually, after crying in front of my spouse, I feel calm and relieved because I just need someone to listen to my feelings. Luckily, he is the type of person who listens to his wife” (W011).

By sharing problems with their spouses, participants were able to relieve stress that they had otherwise kept to themselves. Sharing with and learning from friends or others in their surroundings were also positive steps used to manage emotions. In addition, some participants used social media and internet-based reference sources to search for information on coping strategies for emotional distress:

“I learned from my friends how to reduce my stress and the symptoms of depression and anxiety during pregnancy. My friend advised me to take care of my emotional health and manage my stress level” (W005).

Some participants also relieved emotional distress through spiritual approaches, such as reciting the Quran:

“For me, reciting the Quran was the best form of healing. It really helped me calm my mind. Prayers and reciting the Quran are the most important things for me, and they really help me” (W007).

Most participants described support systems, especially spousal support, as helpful in reducing perinatal depression and anxiety. Some also took positive actions, such as seeking help from HCPs, and used relaxation strategies, such as spiritual practices, to alleviate perinatal depression and anxiety.

### Theme 3: mobilizing needs and support

Perinatal women expressed a need for support to alleviate symptoms of depression and anxiety. Both women and their spouses strongly supported the availability of a mobile application that could provide support during periods of emotional distress. The women provided opinions and suggestions about app approaches and beneficial content that could help users reduce perinatal depression and anxiety symptoms. They agreed that a positive user experience could encourage continued use of the app. Expert-verified content and comprehensive information related to perinatal depression and anxiety symptoms were considered helpful for reducing symptoms.

“For me, the most important thing that needs to be applied when developing an app is authorization from medical professionals, such as doctors from the Ministry of Health in Malaysia. This would help gain users’ trust in using and downloading the mental health app in the future” (W016).

One antenatal woman suggested that a practical guide should include comprehensive content or knowledge on depression and anxiety symptoms and how to manage them. One spouse also suggested that the app should be regularly updated to an improved version. In addition to a good user experience, interactive user interface elements, such as videos, animations, and infographics, could be included to make the app more attractive:

“For spouses, it would be better to add animation, such as cartoons, or videos in the app to attract them to use it. In addition, my spouse does not like reading something too wordy, such as articles” (W014).

In addition to a good user experience and interactive user interface, perinatal women expressed that raising awareness was one advantage of mental health apps that could benefit users:

“At least, when there is an app like this, women can realize, ‘Oh, I experience these symptoms,’ and they can understand what they are feeling right now. Then, they may think that it is normal to have these feelings. When they use the app, they can know that they are stressed or facing a particular problem” (W003).

Spousal involvement was also considered important in reducing perinatal depression and anxiety. Comprehensive app content that includes spousal involvement may raise awareness of mental health. In addition, some perinatal women reported that apps could help protect people with mental illness from being stigmatized and labeled by the community:

“Sometimes, we can use mental health apps if we do not want to meet HCPs face to face, because people around us may stigmatize or label us as crazy if we walk into a psychiatric clinic to attend an appointment. Therefore, it is easier for us to use an app if we are inquiring about something related to mental illness” (W018).

Beneficial content suggested by perinatal women during the interviews included spouses’ roles, psychoeducation, and helpful skills. Most perinatal women and spouses agreed that content on spouses’ roles, such as role lists, communication skills, and problem-solving skills, should be included. Perinatal women agreed that including a list of spouses’ roles in the digital intervention could reduce burden and help address depression and anxiety:

“In the app, it is crucial to include the spouse’s role, especially if the wife is a housewife, because helping them is important for emotional well-being. Sometimes, spouses tend to neglect or not take part in helping their wives with household chores because they are tired and busy with work, and the wife ends up handling everything alone, including the children” (W013).

In addition to lists of spouses’ roles, communication skills between perinatal women and their spouses were considered important for alleviating emotional distress during the perinatal period:

“I like to communicate with my spouse in many ways, but I would be more satisfied if I could communicate face to face. I really need someone to talk with me during my postnatal period” (W017).

Furthermore, most perinatal women expressed that signs and symptoms of depression and anxiety should be emphasized because not all women are aware of these symptoms when they occur:

“I think the content should include the signs and symptoms of depression and anxiety and how to recognize those symptoms when they occur, so this should be explored first” (W002).

Perinatal women also suggested including helpful skills, such as childcare management, perinatal care, and coping mechanisms, in the app because these skills could reduce stress as women adapt to motherhood:

“I think child management is important, especially for first-time mothers. For instance, the app should include how to breastfeed the baby and how to take care of the newborn after childbirth. Those things need to be included because not all mothers have experience handling a baby” (W003).

In addition to childcare management, one postnatal woman’s spouse suggested including information on wound care and a healthy diet for mothers who gave birth by cesarean section. In summary, perinatal women provided suggestions on app characteristics that could attract users, such as a positive user experience, an interactive user interface, and awareness-raising functions that could encourage continued use. They also suggested that mental health app content should include several elements, such as spouses’ roles, psychoeducation, and helpful skills.

## Discussion

In brief, perinatal women and their spouses in this study perceived depression and anxiety as affecting both fetal and maternal health. Analysis of 35 transcripts showed that perinatal women and their spouses experienced emotional, psychological, and social distress during the perinatal period. Social challenges, including LDRs, financial difficulties, workplace stress, child-related responsibilities, and limited spousal support, were perceived as contributing to emotional distress. Participants also described difficulties adapting to parenthood roles and responsibilities during the transition to parenthood. Strong support systems, especially from spouses, family members, and friends, were considered important for reducing emotional distress among perinatal women.

In addition, perinatal women used positive steps and relaxation strategies to reduce stress, including seeking professional assistance, spending time alone, sharing concerns with trusted individuals, and engaging in leisure activities. Spirituality also served as an important culturally relevant coping mechanism for perinatal women and spouses. However, spouses provided less detailed accounts of their coping strategies; their narratives focused more on fulfilling responsibilities and managing family demands than on addressing their own emotional needs. This pattern may reflect broader evidence that men are less likely to disclose emotional difficulties or seek support because of societal expectations surrounding masculinity and caregiving roles [27].

Spousal involvement was particularly valued when partners listened actively, provided reassurance, and shared household responsibilities. Perinatal women who perceive higher levels of spousal support may experience less burden, isolation, and emotional distress; previous research has also shown that greater spousal support is associated with lower levels of perinatal depression and anxiety and better adjustment to motherhood [28]. Although spouses acknowledged their responsibility to support perinatal women, they often framed this role through practical actions, such as helping with household chores, reducing physical workload, and ensuring adequate rest. This may suggest that spouses conceptualize support primarily as instrumental assistance rather than emotional caregiving, consistent with evidence that spouses often express support through tangible caregiving behaviors and practical problem-solving [29]. Although these actions are beneficial, previous research indicates that emotional validation, empathic communication, and psychological availability are also important for protecting maternal mental health.

During the interviews, perinatal women and spouses also shared views on the use of technology to support women experiencing emotional distress. They provided opinions and suggestions on the design and content of a digital intervention to make it attractive and acceptable to users. Because conventional approaches to treating perinatal depression and anxiety may be limited by high dropout rates, low adherence, and stigma-related barriers to help-seeking, participants viewed digital interventions as potentially more accessible and acceptable alternatives [30]. Such interventions may help perinatal women manage depression and anxiety and support emotional well-being. They may also help spouses support perinatal women with depression and anxiety by providing useful content and problem-solving guidance [16,30]. Digital interventions may further facilitate help-seeking by providing information on available mental health services and referral pathways for individuals with more severe symptoms requiring professional intervention.

Some perinatal women appeared to view depression and anxiety symptoms as part of normal perinatal adjustment and therefore did not seek treatment from HCPs. Although evidence indicates that depression and anxiety during the perinatal period can negatively affect fetal development [31-33], perinatal women may not be fully aware of these adverse outcomes and may need greater awareness of their potential consequences. Fellmeth et al. [34] reported low awareness of perinatal depression among antenatal mothers in Delhi and Maharashtra, along with poor public knowledge of mental illness and negative attitudes toward it. This may be because antenatal women are often unaware of common mental illnesses during this period and may be unable to detect them at early stages, which can negatively affect their well-being [35]. According to Sunday et al. [36], poor awareness and treatment of anxiety and/or depression during pregnancy may also be related to women’s reluctance to disclose symptoms, discrimination, stigma, and negative perceptions of people with mental illness.

Perinatal women emphasized LDRs, workplace stress, and lack of social support as major factors contributing to perinatal depression and anxiety. Their spouses also reported that financial constraints and LDRs were triggers for depression and anxiety during the perinatal period. Jahan et al. [37] reported that unwanted or unplanned pregnancies, low socioeconomic status, and lack of perceived social support were major risk factors for depression during the antenatal period. Perinatal women in the present study also acknowledged that spouses play an important role in ensuring that women receive professional help if they experience depression and anxiety during the perinatal period. Similarly, McCarthy et al. [38] reported that antenatal women described poor communication and lack of spousal support as major stressors that can lead to emotional distress during pregnancy.

Perinatal women with depression and anxiety were reluctant to seek help from HCPs because they feared being stigmatized and labeled by the community. Ford et al. [39] reported that perinatal women perceived social pressure and stigma as preventing them from honestly sharing their feelings, leading to shame and anxiety about losing custody of their children. In addition, limited knowledge of perinatal mental health issues can make it difficult for women to identify symptoms and seek treatment. Barriers that prevent perinatal women from seeking help for perinatal psychological distress include difficulty expressing feelings, cultural barriers, lack of knowledge about symptoms of postnatal depression, and the attitudes of family, friends, and HCPs [40]. Most perinatal women prefer to seek help from informal sources, such as spouses, family members, and friends, rather than formal sources such as HCPs [41].

Beerli et al. [42] reported that internet-based therapies may help reduce symptoms in cases of mild to moderate antenatal anxiety and depression. Digital technology can also support targeted communication through health-promotion messages and reminders, provide instant feedback and virtual assistance, improve access to health information, reduce barriers to distance learning, and make better use of existing health resources [43]. Because conventional approaches may be limited by high attrition rates, time constraints, and community stigma, some women in this study agreed that digital approaches for educating themselves and their spouses about depression and anxiety could help reduce symptoms. These findings also suggest that psychoeducation on antenatal anxiety and depression may not have been delivered successfully through conventional approaches [30]. Therefore, digital health technology may be a useful strategy for delivering evidence-based healthcare [44]. Increasing perinatal women’s and spouses’ knowledge and understanding of the risks of anxiety and depression for both mother and child is important. The present study extends previous evidence by identifying the specific content desired by both perinatal women and spouses. These findings suggest that future digital interventions should move beyond symptom screening alone and incorporate spouse-inclusive educational and self-management components.

This study has several limitations. First, the participants were mainly Malay because of the clinic population and attempts to recruit Chinese and Indian participants were unsuccessful because they declined participation. Future studies should aim for greater cultural diversity. Second, the study included only perinatal women and their spouses and did not involve other key individuals, such as family members or friends, who may also influence or support maternal mental health. Third, participants were not recruited as dyads, or matched women and spouses, which limits the ability to directly compare paired responses. Fourth, participation from postnatal women was limited because most did not attend follow-up appointments at the study sites. Future research should consider including government clinics to better capture postnatal cases. Fifth, the sample included more primigravida than multigravida women, which may affect the balance of experiences; future studies should aim for more equal representation of both groups.

In conclusion, analysis of 35 transcripts, including 20 perinatal women and 15 spouses, highlighted the emotional, psychological, and social challenges experienced during the perinatal period. Perinatal women and spouses also emphasized the importance of strong support systems from spouses, family members, and friends in reducing emotional distress. In addition, they reported using positive coping and relaxation strategies, such as seeking social support, engaging in self-care, and practicing spiritual or calming activities, to manage stress during the perinatal period. Both groups supported the use of digital interventions and provided suggestions for design and content that could improve acceptability, mental health support, and effectiveness. These findings contribute to a better understanding of the emotional experiences, support systems, coping strategies, and intervention needs of perinatal women and spouses during the perinatal period.

## Figures and Tables

**Table 1.** Demographic characteristics of perinatal women and spouses (N=35)

Participant IDs	Age (year)	Ethnicities	Educations	Employment status
W001	25	Malay	University	Working
W002	30	Malay	University	Working
W003	31	Malay	Secondary school	Housewife
W004	32	Malay	University	Working
W005	34	Malay	University	Working
W006	28	Malay	University	Working
W007	27	Malay	Secondary school	Housewife
W008	31	Chinese	University	Housewife
W009	33	Malay	University	Working
W010	29	Malay	University	Working
W011	35	Malay	University	Housewife
W012	36	Malay	University	Working
W013	32	Malay	University	Working
W014	35	Malay	University	Housewife
W015	36	Malay	University	Working
W016	36	Malay	University	Working
W017	26	Malay	University	Housewife
W018	27	Malay	University	Working
W019	32	Malay	University	Working
W020	34	Malay	University	Housewife
S001	34	Malay	University	Government sector
S002	29	Malay	University	Government sector
S003	26	Malay	University	Private sector
S004	27	Malay	Secondary school	Self-employed
S005	39	Malay	University	Government sector
S006	37	Malay	University	Private sector
S007	35	Malay	University	Private sector
S008	27	Malay	Secondary school	Self-employed
S009	30	Malay	University	Government sector
S010	33	Malay	University	Private sector
S011	30	Malay	University	Government sector
S012	40	Malay	University	Private sector
S013	32	Malay	University	Government sector
S014	32	Malay	Secondary school	Private sector
S015	28	Malay	University	Government sector

**Table 2.** Comparative thematic findings from interviews with perinatal women and spouses

Themes	Subthemes	Categories	Perinatal women’s perspectives	Spouses’ perspectives
Adjusting to a new period of life	Emotional distress	Not feeling normal	Experienced emotional instability, tearfulness, and personality changes. Reported feeling that life was not normal, becoming more sensitive and irritable, and losing interest in usual activities.	Did not strongly identify personal distress but observed emotional and behavioral changes in their partners, such as talking to themselves and mood swings.
		Mental preoccupation	Frequently experienced overthinking and negative thoughts about childbirth, breastfeeding, and infant care.	Recognized the danger of negative thinking but rarely discussed personal concerns or reported intrusive thoughts of their own.
	Psychological distress	Cognitive functioning	Reported reduced concentration, motivation, and social engagement.	Noticed behavioral changes in their partners but did not elaborate on their own difficulties.
		Physical discomfort	Experienced fatigue, sleep difficulties, and physical discomfort. Women who delivered by cesarean section reported restricted movement as a source of distress.	Recognized physical limitations as contributing to their partners’ distress but rarely described experiencing physical discomfort themselves.
	Social distress	Living with others	Experienced stress related to long-distance relationships, limited support, and workplace demands.	Reported long-distance relationships and financial difficulties as major stressors.
		Parenthood adaptation	Felt unprepared for parenting responsibilities and childcare demands.	Felt uncertain about supporting their partners and caring for the baby.
Dealing with perinatal distress	Support system	Spousal participation	Valued emotional support and practical assistance from spouses, including attentive listening, sharing household chores, and accompanying them to clinic appointments.	Provided practical support, including household chores and listening, despite work- and distance-related challenges.
		Family and friends	Relied on family and friends for emotional and practical support with baby care and household tasks.	Recognized the importance of support from extended family; some relied more heavily on this support rather than taking an active personal role.
	Stress-relieving strategies	Positive steps	Sought professional help, social support, and information resources.	Were less likely to seek help or share emotional concerns.
		Relaxation strategies	Used self-care activities and spiritual practices to reduce stress.	Reported limited use of personal stress-management strategies.
Mobilizing needs and support	App approaches	Good user experience	Preferred trustworthy, accessible, and user-friendly apps that were regularly updated and not excessively text-heavy.	Preferred concise, practical, and regularly updated content, with direct information rather than lengthy descriptive articles.
		Interactive user interface	Favored videos, animations, and infographics.	Supported engaging and interactive multimedia features.
		Awareness raising	Viewed apps as a private source of mental health information.	Viewed apps as tools for monitoring and understanding emotional well-being.
	Beneficial content	Spouses’ roles	Suggested clearer guidance on spouses’ roles and responsibilities.	Suggested guidance on providing effective support.
		Psychoeducation	Requested information on perinatal mental health and help-seeking.	Requested information on symptom recognition and support strategies.
		Helpful skills	Focused on coping strategies, childcare management, and self-care.	Focused on communication skills and ways to support their partners.
